# Hyperspectral screen-image-synthesis meter with scattering-noise suppression

**DOI:** 10.1038/s41598-023-47785-3

**Published:** 2023-11-24

**Authors:** Yeh-Wei Yu, Ming Le, Tsung-Hsun Yang, Cheng-Hsien Chen, Pin-Duan Huang, Chi-Shou Wu, Che-Chu Lin, Tsung-Xian Lee, Ching-Cherng Sun

**Affiliations:** 1 Department of Optics and Photonics, National Central University, Chung-Li, Taoyuan, 320317 Taiwan; 2 Optical Sciences Center, National Central University, Chung-Li, Taoyuan, 320317 Taiwan; 3 Graduate Institute of Color and Illumination Technology, National Taiwan University of Science and Technology, Taipei, 106335 Taiwan; 4 Department of Electrophysics, National Yang Ming Chiao Tung University, Hsinchu, 300093 Taiwan

**Keywords:** Optics and photonics, Optical techniques

## Abstract

The screen image synthesis (SIS) meter was originally proposed as a high-speed measurement tool, which fused the measured data from multiple sample-rotational angles to produce a whole-field measurement result. However, it suffered from stray light noise and lacked the capability of spectrum measurement. In this study, we propose an SIS system embedded with a snapshot hyperspectral technology, which was based on a dispersion image of the sparse sampling screen (SSS). When a photo was captured, it was transformed and calibrated to hyperspectral data at a specific sample-rotational angle. After the hyperspectral data in all sample-rotational angles were captured, an SIS image-fusion process was then applied to get the whole field hyperspectral data. By applying SSS to the SIS meter, we not only create a screen image synthesis hyperspectral meter but also effectively address the issue of stray-light noise. In the experiment, we analyze its correctness by comparing the hyperspectral value with a one-dimensional spectrum goniometer (ODSG). We also show the 2D color temperature coefficient distribution and compare it with the ODSG. Experimental results also demonstrate the feasibility in terms of both spectrum distribution meter and color coefficient temperature distribution meter.

## Introduction

Intensity distribution meters and bidirectional scattering distribution function (BSDF) meters are essential tools in optical design^1–7^, optical modeling^8–14^, and color estimation^15,16^. The conventional technique for intensity distribution measurement, goniophotometer, involves time-consuming point-by-point scanning^15–20^. To overcome this limitation and improve measurement speed, image-based technologies have emerged as promising alternatives^21–27^. Notable high-speed technologies include the image-sphere system^28,29^, conoscopy^30–32^, and the Screen Image Synthesis (SIS) system^33–35^.

The image-sphere system utilizes a camera and a reflective sphere to capture the emitted light from a source^28,29^. By scattering the light using the reflective sphere, the system obtains a snapshot of the light distribution in all directions. However, the fixed size of the sphere restricts the far-field distance and limits the sample size. Moreover, the scattering of light from the spherical surface introduces two types of stray-light noise^36^. First, scattering from one part of the spherical surface tends to project onto other parts, leading to cross-talk noise. Second, scattering from one part of the spherical surface can hit the sample and reflect onto other parts, resulting in sample-reflecting-stray-light (SRSL) noise. Conoscopy, on the other hand, is a microscope-based technique that captures the intensity distribution of a small light source within a single plane^30–32^. A camera records the light distribution, providing a snapshot of the source. However, suppressing SRSL noise in conoscopy is a complex task that often requires specific lens design, coatings, and immersion oil operation.

In contrast, the SIS system has demonstrated its ability to measure intensity distribution as well as BSDF^33–35^. This system employs a screen and a camera to capture photos as the sample rotates at different angles, generating a whole-field measurement result. The adjustable distance between the screen and the sample offers flexibility in sample size, making the SIS system versatile and suitable for a wide range of samples and applications. The flat screen of the SIS system minimizes cross-talk noise. However, SRSL noise remains considerable due to the large solid-angle occupancy of the screen.

Furthermore, the conventional SIS system lacks spectral or color information. It makes measurement errors occurred by the Metamerism due to the spectral distribution of light source. To address this limitation, we replace the original screen of the SIS system with a black screen containing an array of holes. This modification effectively suppresses SRSL noise and serves as a sparse-sampling screen (SSS). Additionally, a grating is placed in front of the camera, working together with the SSS to enable snapshot hyperspectral technology. It is equivalent to embed an integral field spectroscopy (IFS) snapshot hyperspectral technique inside the SIS system^37–40^.

Another solution could be using a compact snapshot hyperspectral imaging module rotating around the sample to gather all angular responses from the sample. It may leverage advance snapshot hyperspectral solutions^41^, such like the code-aperture technique^42–45^, or the Chip-side technology^46–52^. However, because the intensity distribution measurement needs to measure in far-field distance, the compact size of the input plane means its angular coverage is small. It makes whole-field measurement a time-consuming process. As a result, the SIS with aid of snapshot hyperspectral provides a better solution. It can capture large angle coverage and get rid of SRSL noise at the same time.

In this paper, we successfully develop an SIS hyperspectral (SISH) meter and demonstrate it experimentally. It can benefit color science^53–55^, material identification^56,57^, and optical engineering^58,59^. Because of the sparse-sampling essence, it is compatible with data stream improvement techniques such as compressed sensing^60–63^ or deep-learning approaches^64,65^.

## SIS hyperspectral system

**Figure 1:**
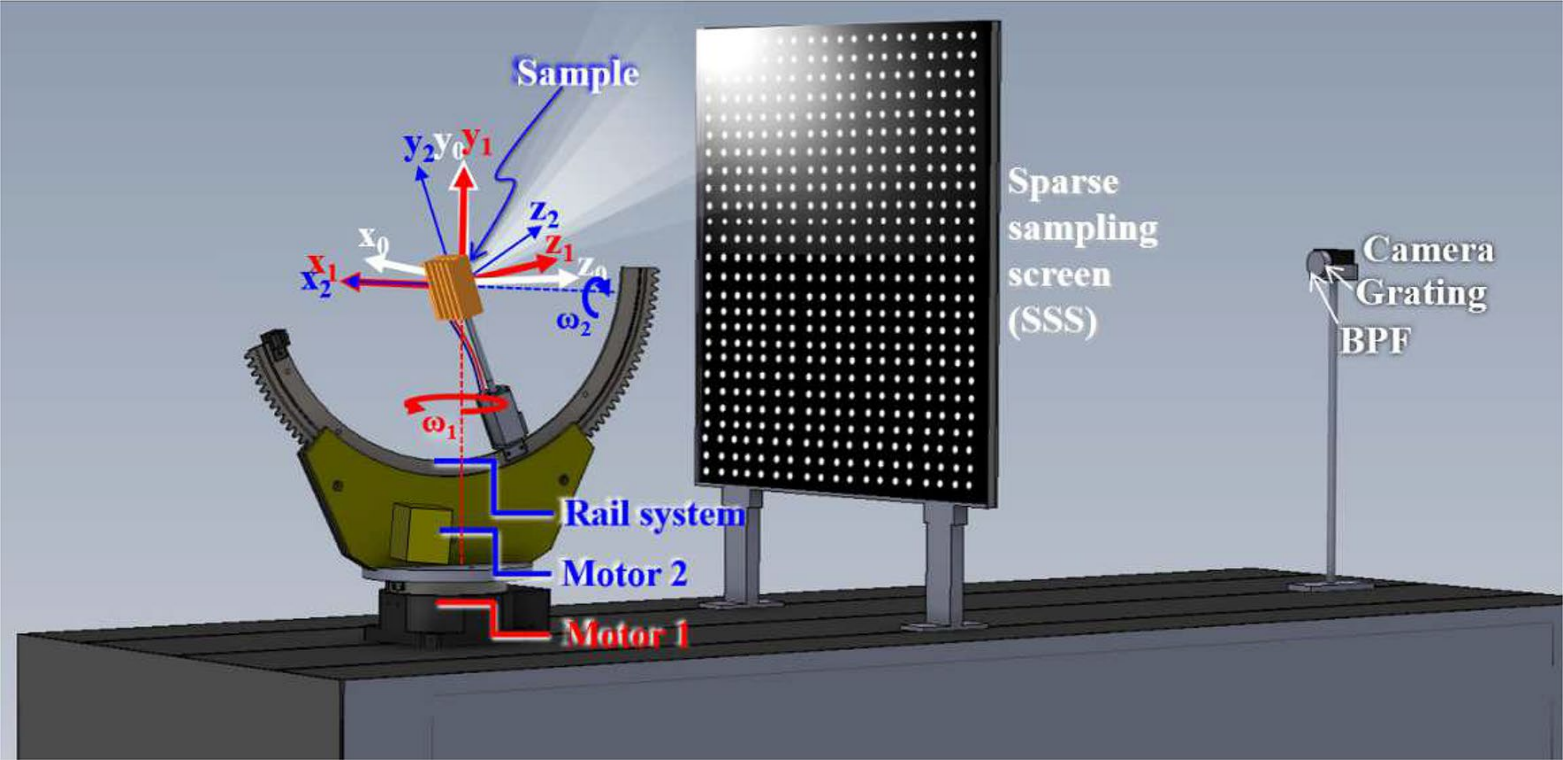
The architecture of the SIS hyper-spectrum system is composed with a sample-rotational system and a snap-shot hyperspectral system. It captures the spectrum distribution with $4\pi$ solid angle.

Figure 1 depicts the SISH system is composed by a snap-shot hyperspectral system and a sample-rotational system. The snap-shot hyperspectral system is composed by a camera, a grating, a band pass filter (BPF) and a sparse-sampling screen (SSS). The irradiance captured by the CMOS sensor corresponded to the radiance from each position of the screen and was captured by the camera. The sample rotational system is composed by Motor1, Mortor 2, a rail system and a sample mount. A Cartesian coordinate system $(x_0, y_0, z_0)$ is defined in the snap-shot hyperspectral system. The sample-rotational system is driven using two motors. Motor 1 rotates all components of the sample-rotational system along the red axis. A rail system is fixed on the loading side of Motor 1 and is driven by Motor 2, which rotates the load components along the blue axis. $(x_1, y_1, z_1)$ is the cartesian coordinate system defined on the loading side of Motor 1. When Motor 1 rotates, all the components loaded by Motor 1 rotate $\omega _1$ along the $y_0$-axis. $(x_2, y_2, z_2)$ is the cartesian coordinate system defined on the loading side of the rail system. When Motor 2 rotates, the sample rotates $\omega _2$ along the $x_1$-axis. Motor 1 rotates from $-66^\circ$ to $66^\circ$ with a step of $33^\circ$. Motor 2 rotates from -150$^\circ$ to 180$^\circ$ with a step of 30$^\circ$. The two motors rotate to 60 angles to cover the $4\pi$ solid angle surrounding the sample. After the progress of parameter optimization and system calibration, the image reconstruction algorithm is used to obtain the 2-D light distribution.

### Snap-shot hyperspectral technique

**Figure 2:**
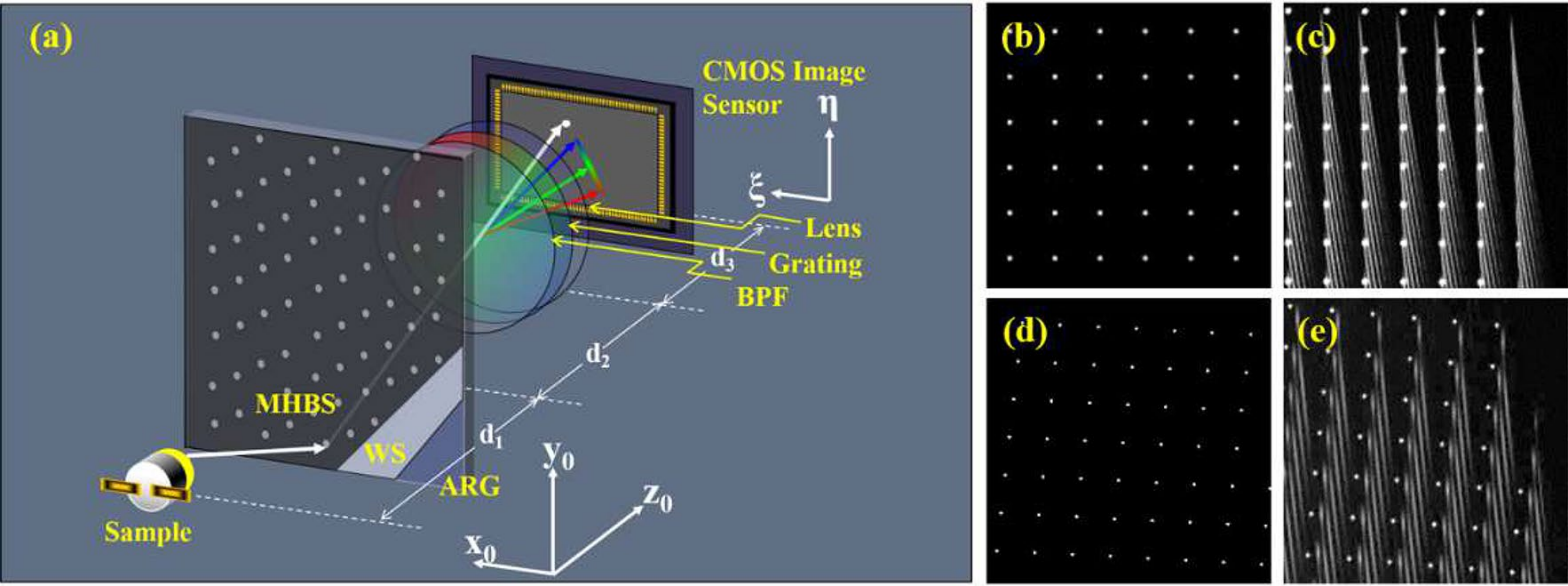
(**a**) The system setup of the snap-shot hyperspectral; (**b**) the holes distribute in square grid; (**c**) the dispersion light of the square-grid holes overlaps to each other; (**d**) the improved holes distribution; (**e**) the dispersion light of the improved holes was separated from each other.

Figure 2a shows the schematic diagram of the snap-shot hyperspectral system. The camera consists of a CMOS image sensor and a lens, and it forms the image of the SSS on the CMOS image sensor. A grating mounted in front of the lens is used to disperse the image. The SSS is used to depress the SRSL noise and to perform the spectrum acquiring by Spatial multiplexing. The SSS is composed by a multi hole black stick (MHBS), a white stick (WS), and an AR coating glass (ARG) with size 601 (H) $\times 496$ (W) $\times 3$ (D) (mm$^3$). The optical property of the WS is scattering with Lambertian distribution. The band pass filter (BPF) in front of the grating limits the detection band within 390–710 nm, to remove the noise caused by field curvature aberration. The material of both the MHBS and WS are polyvinyl chloride (PVC). The distance between each hole is 10mm, and the hole diameter is 0.8mm. It makes the scattering reflection of the screen decreasing to $0.6\%$. The SRSL noise is depressed accordingly. Figure 2b shows a design of the square holes array. The total number of holes in the MHBS is 2, 401. However, series distortion aberration of the off-axis dispersion image makes the spectrum image banding. The neighboring first-order spectrums overlap with each other, as shown in Fig. 2c. The spectrum detection of these off-axis holes thus failed. To improve the overlapping problem, we calculate the gradient of all the first order spectrum and increase the distance between the holes along the orthogonal direction of the gradient. Figure 2d shows the new design. Because of the distance increase, the total area of the holes is expanded. Some of the holes in the new design are located outside the SSS and are removed. So, the total number of the holes in the new design decreases to 2233. Figure 2e shows the dispersion image of the new design, and the off-axis spectrum overlapping problem are resolved.

For the imaging system shown in Fig. 2a, the object distance is $d_2$, and the image distance is $d_3$ (as shown in Eq. (1). The light beam dispersed by the grating with the dispersion angle $\Delta \theta (\lambda )$. The corresponding shifting of the dispersed point is

1 $$\begin{aligned} \Delta r(\lambda )=d_3\cdot \Delta \theta (\lambda ). \end{aligned}$$

In the linear space invariant region of the image system, the spectrum can be treated as a convolution of the image of the hole and the spectrum curve. Thus, the spectral resolvance (V) is limited by the hole size, and is written as

2 $$\begin{aligned} V=\frac{d_2\cdot [\Delta \theta (\lambda _{max})-\Delta \theta (\lambda _{min})]}{H}. \end{aligned}$$

The designed spectral resolution (R) is calculated from the spectral resolvance

3 $$\begin{aligned} R=\frac{\lambda _{max}-\lambda _{min}}{V}. \end{aligned}$$

It shows smaller hole size and longer object distance ($d_1$) lead to higher spectral resolvance. In this paper, the CMOS image sensor has 5 million pixels and the pixel pitch are $3.45 \, \upmu$m; the lens has focal length 5 mm; $d_2=750$ mm; $d_3=5.03$ mm. The image of the hole occupies $3\times 3$ pixels in the CMOS Image Sensor. The designed spectral resolution is 5 nm. The distance $d_1$ between the sample and the *SSS* is 500 mm, and the longest distance between the sample and the diagonal edge of the *SSS* is 612 mm. Therefore, the angular resolution is $0.9^\circ \sim 1.2^\circ$.

### Spectrum retrieval process

$SSS(x_0, y_0; \lambda )$ is the hyperspectral data on *SSS*, and it is retrieved from a 2D photo $G(\xi ,\eta )$, where $(x_0, y_0)$ is the position of the sampling hole on *SSS*, and $(\xi ,\eta )$ is the coordinate of the CMOS image sensor. The point spread function of the optical system comparing to the size of the sampling hole is small enough, and the location $(\xi ,\eta )$ of the dispersed image for the sampling hole located at $(x_0, y_0)$ is expressed as a quadratic function of $\lambda$. The relation between the 2D photo and the hyperspectral data can be expressed as

4 $$\begin{aligned} \left[ \begin{array}{c} \xi \\ \eta \end{array} \right] =\left[ \begin{array}{ccc} a(x_0,y_0) &{} b(x_0,y_0) &{} c(x_0,y_0) \\ d(x_0,y_0) &{} e(x_0,y_0) &{} f(x_0,y_0) \end{array} \right] \left[ \begin{array}{c} \lambda ^2 \\ \lambda \\ 1 \end{array} \right] \end{aligned}$$

We use a laser beam with wavelengths of 457nm, 532nm, and 671nm to pass through a diffuser sequentially. The scattering light illuminated the SSS. And we capture the dispersed image of SSS to get $G_r(\xi ,\eta )$, $G_g(\xi ,\eta )$, and $G_b(\xi ,\eta )$. Because the three dispersed images are discrete points array. It is easy to measure the position $(\xi ,\eta )$ corresponding to each sampling hole located at $(x_0, y_0)$. Use the three photos, we have 6 equations to solve the coefficients, i.e., $a(x_0, y_0)\sim f(x_0, y_0)$. Then, we can transform a captured photo $G(\xi ,\eta )$ to the hyperspectral raw data

5 $$\begin{aligned} SSS_0(x_0,y_0;\lambda )=G(\xi (x_0,y_0;\lambda ),\eta (x_0,y_0;\lambda )). \end{aligned}$$

In the calibration process, we used a white light LED as the standard light source. We take a picture and calculate hyperspectral raw data $SSS_w (x_0,y_0;\lambda )$ using Eq. (5). Then we shifted a spectrometer (ISUZU OPTICS, ISM-Lux, corrected by ITRI-CMS) two-dimensionally to measure the spectrum distribution, i.e., $SSS_t (x_0,y_0;\lambda )$ . Thus, the calibration function was

6 $$\begin{aligned} C (x_0,y_0;\lambda )=\frac{SSS_t(x_0,y_0;\lambda )}{SSS_w(x_0,y_0;\lambda )}. \end{aligned}$$

Therefore, when the hyperspectral meter was applied to measure an arbitrary light source, the calibrated hyperspectral was:

7 $$\begin{aligned} SSS (x_0,y_0;\lambda )=SSS_0(x_0,y_0;\lambda )\cdot C(x_0,y_0;\lambda ). \end{aligned}$$

### Image reconstruction algorithm of the SISH system

Because the imaging system is static, the sample should be rotated in steps to fill all detection angles. The rotational angles of Motor 1($\omega _1$) and Motor 2 ($\omega _2$) are expressed as functions of integer indices i and j respectively,

8 $$\begin{aligned} \omega _1= & {} 33\cdot (i-3), \end{aligned}$$

9 $$\begin{aligned} \omega _2= & {} 33\cdot (j-3), \end{aligned}$$

When the sample rotates, we need to map detected hyperspectral distribution on the screen in Cartesian coordinate, i.e. $SSS(x_0,y_0;\lambda )$, to the captured hyperspectral data with sample-rotational angle in spherical coordinate, i.e. $SSS_{i,j}(\theta ,\phi ;\lambda )$. The Cartesian coordinates attached to the screen, Motor 1, and Motor 2 (as well as samples) are expressed as the span of the bases $B_0$, $B_0$, and $B_1$, i.e., $$B_0\left[ \begin{array}{ccc}x_0&y_0&z_0\end{array}\right] ^T$$, $$B_0\left[ \begin{array}{ccc}x_1&y_1&z_1\end{array}\right] ^T$$, and $$B_1\left[ \begin{array}{ccc}x_2&y_2&z_2\end{array}\right] ^T$$, respectively. The origin of the coordinate frames is located at the rotational center of the sample. For the rotation of Motors 1, and 2, the rotational matrix is expressed as follows:

10 $$\begin{aligned} \left[ {\mathbb {R}}^1\right] _{B_0}=\left[ \begin{array}{ccc} \cos {\omega _1} &{} 0 &{}\sin {\omega _1}\\ 0 &{} 1 &{} 0\\ -\sin {\omega _1} &{} 0 &{} \cos {\omega _1} \end{array} \right] , \end{aligned}$$

and

11 $$\begin{aligned} \left[ {\mathbb {R}}^2\right] _{B_1}=\left[ \begin{array}{ccc} 1 &{} 0 &{} 0\\ 0 &{} \cos {\omega _2} &{} -\sin {\omega _2}\\ 0 &{} \sin {\omega _2} &{} \cos {\omega _2} \end{array} \right] , \end{aligned}$$

where the subscripts B0 and B1 indicate that they are used as the basis of the corresponding rotational matrices. Using Eqs. (10) and (11), the intensity distribution captured with the Cartesian coordinates attached to the screen is transferred to the Cartesian coordinates attached to the sample^35^

12 $$\begin{aligned} \left[ \begin{array}{c} x_2 \\ y_2 \\ z_2 \end{array} \right] =\left[ {\mathbb {R}}^1\right] _{B_0} \left[ {\mathbb {R}}^2\right] _{B_1} \left[ \begin{array}{c} x_0 \\ y_0 \\ z_0 \end{array} \right] \end{aligned}$$

**Figure 3:**
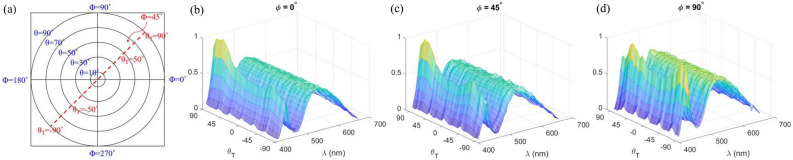
(**a**) The coordinates in the Azimuth equidistant projection and the coordinate for the one-dimension expression, and (**b**–**d**) the hyperspectal of a bias hemisphere package LED measured by the SISH along the azimuth angle $\phi =0^\circ$, $45^\circ$, and $90^\circ$, respectively.

The Cartesian coordinates are mapped to spherical coordinates (Fig. 3) as follows:

13 $$\begin{aligned} \theta= & {} tan^{-1}\frac{\sqrt{x_0^2+y_0^2}}{z_0}, \end{aligned}$$

14 $$\begin{aligned} \phi= & {} tan^{-1}(\frac{y_0}{x_0}), \end{aligned}$$

After the images for different rotational angles are captured at specific $\omega _1$ and $\omega _2$, Eqs. (4)–(7) is used to calculate the hypectral $SSS(x_0,y_0;\lambda )$. Subsequently, Eqs. (8)–(14) are used to calculate the hyperspectral $SSS_{i,j}(\theta ,\phi ;\lambda )$. Where, $\theta$ and $\phi$ is the zenith angle and the azimuth angle of the spherical coordinate, respectively. In case of a forward illumination sample, 30 pictures with various values of $\omega _1$ and $\omega _2$ are used to reconstruct the whole field hyperspectral data. This is expressed as follows:

15 $$\begin{aligned} I_{SIS}(\theta ,\phi ;\lambda )=\sum _{i=1}^5\sum _{j=1}^6\frac{SSS_{i,j}(\theta ,\phi ;\lambda )}{W(\theta ,\phi )}, \end{aligned}$$

where $W(\theta ,\phi )$ is the number of repetitions of the superposition process. Equation (15) means $SSS_{i,j}(\theta ,\phi ;\lambda )$ in all rotational angles are summed, and is divided by $W(\theta ,\phi )$. The calculation of $W(\theta ,\phi )$ uses the same simulation codes as $SSS_{i,j}(\theta ,\phi ;\lambda )$. We replaced $SSS(x_0,y_0;\lambda )$ with an all-ones matrix, where all entry is equal to one. The all-ones matrix is mapped to the spherical coordinate using Eqs. (8)–(12) to produce $OCP_{i,j}(\theta ,\phi )$, which is the area occupied by $SSS_{i,j}(\theta ,\phi ;\lambda )$. Then we sum $OCP_{i,j}(\theta ,\phi )$ in each shot to calculate the number of repetitions

16 $$\begin{aligned} W(\theta ,\phi )=\sum _{i=1}^5\sum _{j=1}^6 OCP_{i,j}(\theta ,\phi ), \end{aligned}$$

Table 1 shows the descriptions of functions used for the hyperspectral retrieval and the SIS fusion. The hyperspectral retrieval process uses the captured photo $G(\xi ,\eta )$ to calculate $SSS(x_0,y_0;\lambda )$, which is the hyperspectral data on the SSS screen. The SIS fusion process maps $SSS(x_0,y_0;\lambda )$ to spherical coordinate with considering the rotational angle of the sample $\omega _1(i)$ and $\omega _2(j)$, and gets $SSS_{i,j}(\theta ,\phi ;\lambda )$. Then all $SSS_{i,j}(\theta ,\phi ;\lambda )$ are fused to get $I_{SIS}(\theta ,\phi ;\lambda )$.

**Table 1:** The function list. .

	Function	Description
Hyperspectral retrieval	$G(\xi ,\eta )$	Captured photo
	$SSS_W(x_0,y_0;\lambda )$	Hyperspectral raw data of a standard light source
	$SSS_t(x_0,y_0;\lambda )$	Hyperspectral raw data of a standard light source measured by a standard spectrometer
	$C(x_0,y_0;\lambda )$	Clibration function
	$SSS(x_0,y_0;\lambda )$	Hyperspectral data calculated from $G(\xi ,\eta )$ in Cartesian coordinate
SIS fusion	$\omega _1(i),\omega _2(j)$	The rotational angle of motor1 and motor2
	${\mathbb {R}}^1,{\mathbb {R}}^2$	The rotational matrix of motor1 and motor2
	$SSS_{i,j}(\theta ,\phi ;\lambda )$	Hyperspectral data calculated from $G(\xi ,\eta )$ in spherical coordinate
	$OCP_{i,j}(\theta ,\phi )$	The area occupied by $SSS_{i,j}(\theta ,\phi ;\lambda )$
	$W(\theta ,\phi )$	The number of repetitions
	$I_{SIS} (\theta ,\phi ;\lambda )$	Whole field hyperspectral data

## System performance estimation methods

The distribution of coefficient of color temperature (CCT) is the key to photometry measurements, and it is convenient to show the whole measurement result using 2D Azimuth equidistant projection. So, we not only estimate the system performance of hyperspectral measurement, but also use the CCT distribution to estimate the system performance. The normalized correlation coefficient (NCC) is used to quantify the system performance of the hyperspectral measurement^66^. The NCC is defined as

17 $$\begin{aligned} NCC=\frac{\sum _{i=1}^m[I_{SIS}(\lambda _i)-{\bar{I}}_{SIS}]\cdot [I_{ODG}(\lambda _i)-{\bar{I}}_{ODG}]}{\sqrt{\sum _{i=1}^m[I_{SIS}(\lambda _i)-{\bar{I}}_{SIS}]^2\cdot \sum _{i=1}^m[I_{ODG}(\lambda _i)-{\bar{I}}_{ODG}]}} \end{aligned}$$

where $I_{SIS}$ and $I_{ODG}$ are the spectrum measured by the SISH and the one-dimensional spectrum goniometer (ODSG), respectively. ${\bar{I}}_{SIS}$ and ${\bar{I}}_{ODG}$ are the mean values of the $I_{SIS}$ and the $I_{ODG}$, respectively. m is the spectrum band number, and the value of m is 55 in this paper. For the performance of the color distribution measurement, we need to calculate the CCT distribution and the color coordinate. According to McCamy formula^67^,

18 $$\begin{aligned} n=\frac{c_x-0.3320}{c_y-0.1858}, \end{aligned}$$

where $c_x$, and $c_y$ are corresponding color coordinate in CIE 1931 x–y chromaticity coordinate system^68^. And then calculate the CCT using the cubic polynomial approximation^69^

19 $$\begin{aligned} CCT=-449n^3+3525n^2-6823.3n+5520.33, \end{aligned}$$

Finally, we use the average of the different (AD) and the different standard deviation (DSD) to evaluate the CCT measurement performance. Different from the unitless NCC, which is used to evaluate the similarity of two curves. The AD and the DSD in unit of color temperature (k) bring the sense of color accuracy and color precision. The AD is defined as

20 $$\begin{aligned} AD=\frac{\sum _{i=1}^N[F_{SIS}(\theta _T)-F_{ODG}(\theta _T)]}{N}, \end{aligned}$$

Where N is the total amount of the measurement of $\theta _T$. $F_{SIS}$ is the CCT distribution or the color coordinate distribution based on the SISH and $F_{ODG}$ is the CCT distribution or the color coordinate distribution based on the ODSG.

21 $$\begin{aligned} DSD=\sqrt{\frac{\sum _{i=1}^N\{[F_{SIS}(\theta _T)-F_{ODG}(\theta _T)]-AD\}^2}{N}}, \end{aligned}$$

**Figure 4:**
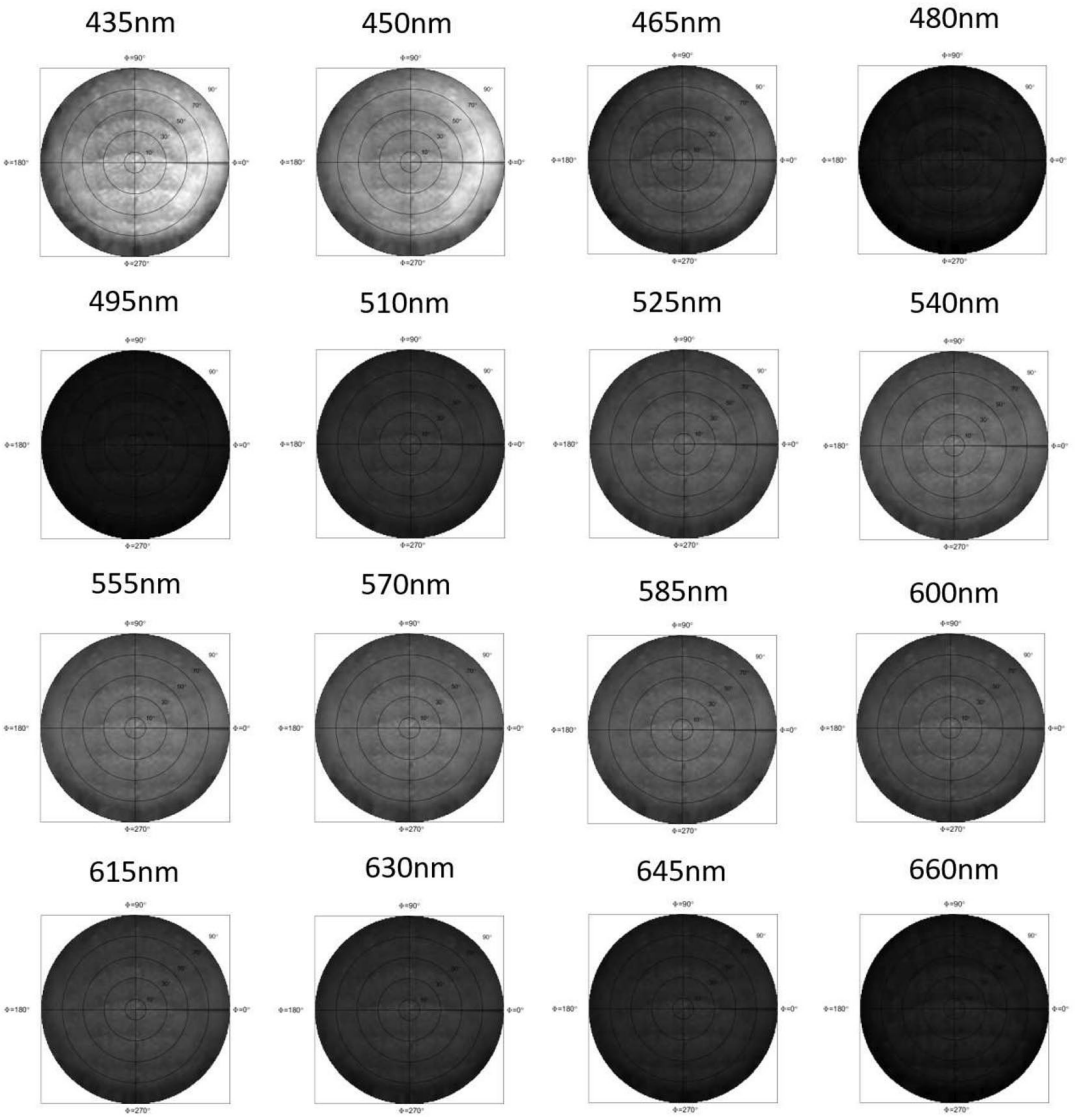
The per-three-band image shows the intensity distribution of different wavelength.

**Figure 5:**
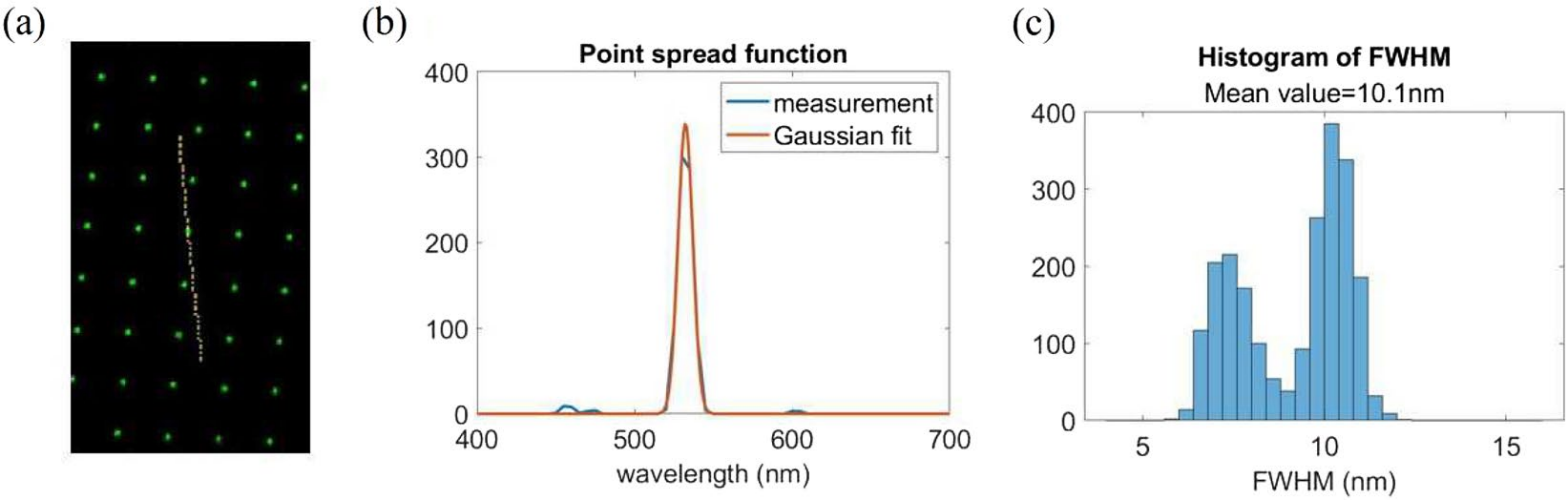
The measurement of the spectral resolution, (**a**) the captured image; (**b**) the point spread function of wavelength; (**c**) the statistical result of FWHM over total 2233 points.

## Experiment and performance evaluation

In the experimental process, we capture the spectrum distribution of different samples by using the SIS with aid of SSS, as shown in Fig. 1. In each rotational angle $(\omega _1,\omega _2)$, a 2D photo $G(\xi ,\eta )$ was taken. Then, a calibrated hyperspectral $SSS(x_0,y_0;\lambda )$ was calculated by putting $G(\xi ,\eta )$ into Eqs. (4)–(7). It was then mapped to the spherical coordinate and was expressed as $SSS_(i,j) (\theta ,\phi ;\lambda )$ by using Eqs. (8)–(12). We count the number of repetitions $W(\theta ,\phi )$ in each position, and then apply these variables to Eq. (15) to calculate $I_{SIS} (\theta ,\phi ;\lambda )$. The calculation software is MATLAB. For sake of clear expression, we draw $I_{SIS} (\theta ,\phi ;\lambda )$ on a disc using the azimuth equidistant projection, as shown in Fig. 3. The direction of $(\theta , \phi ) = (0, 0)$ is normal to the samples. In the experiment, since the measuring angular resolution is $0.9^\circ \sim 1.2^\circ$. We interpolate the measurement result to the spherical coordinate of $( \theta , \phi )$ grid with the angular resolution $1^\circ$. The calculation results produce a total of $180\times 180$ spectrum curves. Figure 3b–d show we extract spectrum distributions $I_{SIS} (\theta _T,0;\lambda )$, $I_{SIS} (\theta _T,45;\lambda )$, and $I_{SIS} (\theta _T,90;\lambda )$ from $I_{SIS} (\theta ,\phi ;\lambda )$. For a specific azimuth angle $\phi$ and $\phi +180^\circ$, the coordinate $\theta _T$ is used to combine the $\theta$ distribution of two specific azimuth angles, $\phi$ and $\phi +180^\circ$, to a single axis. Thus, the angle $(\theta _T, \phi )= (-90^\circ , \phi ^\prime )$ denotes the spherical coordinate $(\theta , \phi )= (90^\circ , \phi ^\prime +180^\circ )$. Because we used LED light sources as the samples, there is almost no backward light, and we only need to discuss the intensity distribution with $|\theta _T |<90^\circ$. In Fig. 3b–d, the sample is a Lab-packaged LED using hemisphere package. The color distribution of LEDs in “Hemisphere” package should be radially symmetrical. However, we deliberately make a “Bias Hemisphere” package to change the color distribution. As a result, the spectrum distributions for the three figures are obviously different. Figure 4 shows the per-three-bands image of the “Bias Hemisphere” package. The blue band (435–460nm) is obviously affected by the bias package, but other bands appear to be unaffected. Because the blue light is emitted by a small die and passes through the phosphor, the smaller etendue makes it easier to be shaped by the hemispherical package lens. Other wavelength bands are re-emitted by the phosphor. The large etendue makes it hard to be shaped by the hemispherical package lens.

In order to evaluate the system performance, we used a laser beam with wavelength 532nm to illuminate a diffuser and to capture the image $G(\xi ,\eta )$. A smoothing process was applied to average each pixel with 8 surrounding pixels, to get a new value of the pixel. The image after smoothing process is shown in Fig. 5a. It shows the hole distribution is sparse enough to separate the spectrum of each hole and makes sure the angular resolution follows the design value, i.e., $0.9^\circ$ to $1.2^\circ$. However, the continuous temporal spectrum makes the image of the hole of different wavelength overlapping each other, the spectral resolution should be evaluated by the full width of half maximum (FWHM) of the point spread function (PSF) of the wavelength. The spectrum distribution corresponding to a hole location $(x_0,y_0)$ is calculated by Eq. (5). Thus, the path of a spectrum distribution in the photo $(\xi (x_0,y_0;\lambda ),\eta (x_0,y_0;\lambda )$ is illustrated with a yellow dash line in the figure. The PSF of the wavelength can be measured along the spectrum path, such as the blue curve in Fig. 5b. We apply Gaussian fitting with $95\%$ fitting confidence, and get the orange curve. The full width of half maximum (FWHM) of the fitted Gaussian function is calculated accordingly. Figure 5c shows the statistic FWHM over total 2233 points. Because the field-curvature aberration is induced by the camera lens, the on-focusing depth changes with (x0, y0). It makes the FWHM varies from 5.6 to 12 nm, and the average FWHM is 10.1 nm. It turns out as the spectral resolution of the experimental results, and is about twice the theoretical spectral resolution calculated by Eq. (3).

**Figure 6:**
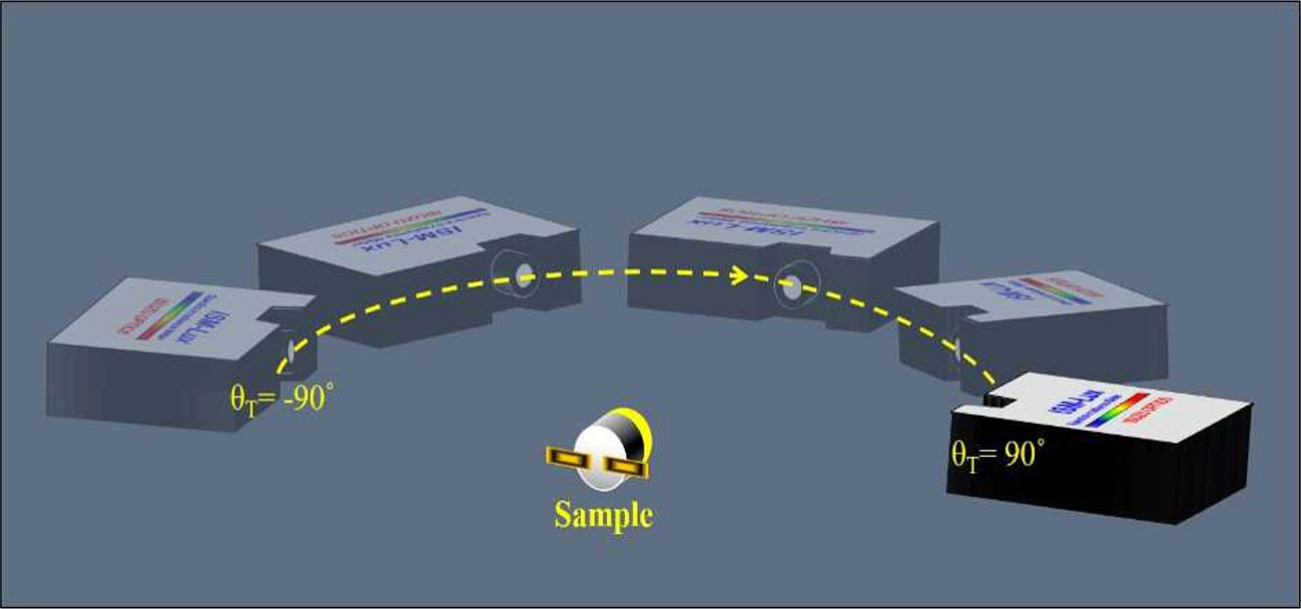
The one-dimensional spectrum goniometer (ODSG).

We used the spectrum meter (ISUZU OPTICS, ISM-Lux, corrected by ITRI-CMS) as a standard device to build up an ODSG. It rotates around the sample to measure the comparison spectrum $I_{ODG}(\lambda )$ of the sample per 2 degrees, as shown in Fig. 6.

We tested four samples with SISH system and the ODSG. The four samples are Lab-packaged LEDs in the form of “Bias Hemisphere”, “Hemisphere extended height coating (Hemisphere EHC)”, “Conformal without lens” and “Cup”. Where, “cup” and “Conformal without lens” were the conventional techniques to achieve uniform CCT distribution^67^. The “Hemisphere EHC” was then proposed to achieve better uniformity of CCT distribution^70^. However, to show the value of SISH meter, which can be applied to check the quality of design or manufacture, we deliberately change the package parameter. For the Lab-packaged “Bias Hemisphere”, we made the lens biasing from the center, to simulate a poor package quality. For the Lab-packaged “Hemisphere EHC”, we deliberately choose the extended height of 1mm to produce poor CCT uniformity.

**Figure 7:**
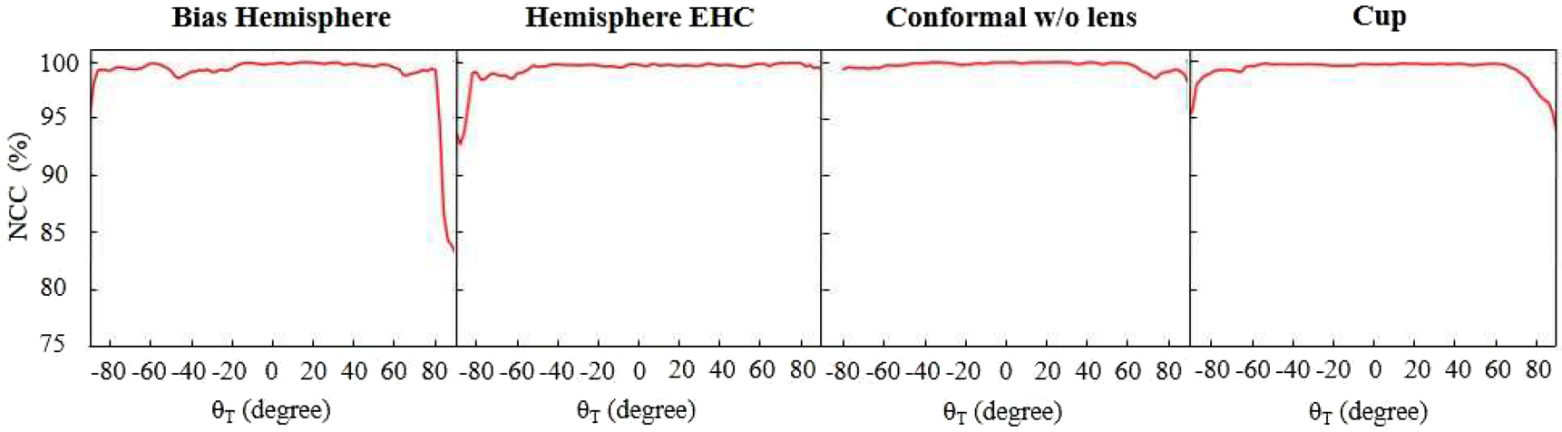
The NCC value between the $I_{ODG}(\lambda )$ and the $I_{SIS} (\lambda _i )$ for $\phi =0^\circ$.

In the experiment, $I_{ODG}(\lambda )$ for $\phi =0^\circ$were taken by the ODSG, and $I_{SIS} (\lambda _i )$ for $\phi =0^\circ$were taken by the SISH meter. Then we calculate the NCC values between the measured results of the $I_{ODG}(\lambda )$ and the $I_{SIS} (\lambda _i)$. Figure 7 shows for all types of packages, in the detection region $|\theta _T |<80^\circ$, NCC values are higher than $95\%$. The system performance gets worse in large $\theta _T$ because the intensity in large $theta_T$ are weak. It induces noise and degrades the NCC values. Especially, the package of “Bias Hemisphere” in the region $\theta _T>80^\circ$, weak signal and dramatic spectrum variation lead to the drop of NCC. We also need to note int the package of conformal without lens, the signals with $\theta _T<80^\circ$ are so weak, such that the ODSG detects only the noise. It makes the comparison values $I_{ODG} (\lambda _i )$ within these regions not trustable. So, we removed all the system evaluation within this region.

**Figure 8:**
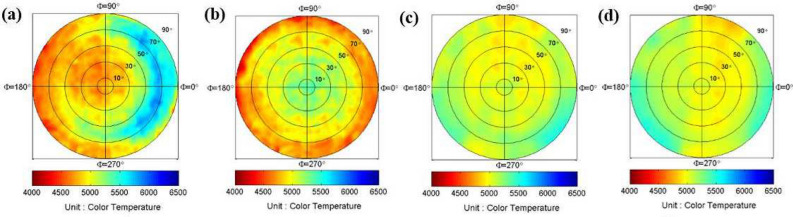
The 2D Azimuth equidistant projection of CCT.

Because a single value CCT is calculated from the 1D spectrum, the 3D hyperspectral data can be transformed to the 2D CCT distribution, and thus be shown in a single 2D picture. And it is sensitive to spectrum variation. Besides, color distribution is an important parameter for the lighting and display manufacturing industries. So, we used Eqs. (18) and (19) to calculate the spatial distribution of the CCT. Figure 8 shows the 2D Azimuth equidistant projection of CCT, and it was used to compare the CCT uniformity of different packages. Although the “Hemisphere EHC” was reported as owning the best CCT uniformity^71^. Figure 8 shows “conformal w/o lens” and “cup” have better CCT uniformity. It is because the performance of uniformity highly depends on package parameters and package quality. We deliberately change the package parameter to degrade the CCT uniformity of “Bias Hemisphere” and “Hemisphere EHC”. The package of “Bias Hemisphere” shows the CCT distribution of higher CCT in the right side, as shown in Fig. 8a. Since the short wavelength contributes to higher CCT, this picture is coincident with the wavelength distribution as shown in Fig. 3b–d.

**Figure 9:**
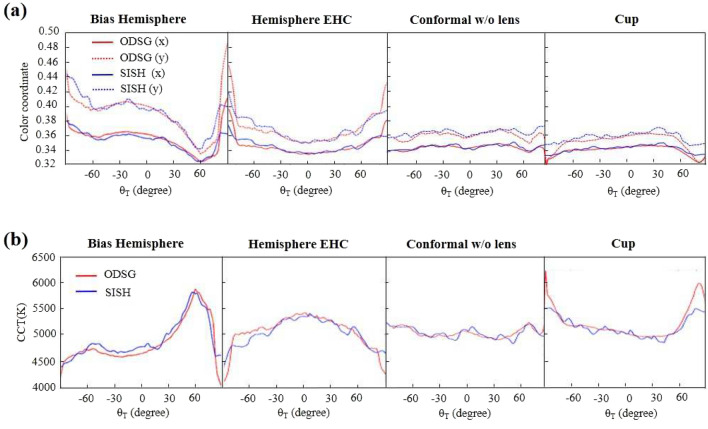
The comparison of (**a**) color coordinates, and (**b**) CCT.

Figure 9a shows the color coordinate measured along the angle $\phi =0$. Where the red curves are measured by the ODSG, and the blue curves are measured by the SISH. It shows most SISH curves are coincident with the ODSG curves within the detection region $|\theta _T |<80^\circ$, except some regions within $60^\circ<|\theta _T |<80^\circ$. The errors are large inaccuracy induced by the weak irradiance, as $|\theta _T|$ gets larger. Figure 9b shows the calculated CCT, based on the color coordinates. Therefore, the mismatch between the ODSG and the SISH appears in the similar regions of Fig. 9a. Table 2 shows the AD and the DSD calculated from the CCT curves within $|\theta _T |<60^\circ$. It shows the maximum value of |*AD*| and |*DSD*| are smaller than 69k.

**Table 2:** The AD and the DSD calculated from the CCT within $|\theta _T |<60^\circ$. .

	Bias hemisphere	Hemisphere EHC	Conformal w/o lens	Cup
AD	−68.6K	52.0K	5.1K	30.8K
DSD	65.7K	52.6K	54.8K	50.5K

## Conclusion

The lighting and display manufacturing industries need high throughput measurement in either color accuracy or spectrum distribution. The SIS meter was proposed to make high-speed whole-field light-distribution measurement. However, it suffered from stray-light noise and lacked the capability for spectrum measurement. We proposed a Snapshot Hyperspectral technology to build up a SISH. The screen of the SISH was replaced by SSS. There are totally 2233 holes in the SSS, and was used to sample the intensity distribution sparsely. It makes the scattering reflection of the screen decreasing to $0.6\%$, and the SRSL noise is depressed accordingly. In the proposed snap-shot hyperspectral, the designed spectral resolution is 5nm, and the designed angular resolution is $0.9^\circ$ to $1.2^\circ$.

In the spectrum retrieval process, the laser beams with wavelengths 457 nm, 532 nm, and 671 nm were used as light sources to get the dispersed images of SSS, i.e., $G_r (\xi ,\eta )$, $G_g (\xi ,\eta )$, and $G_b (\xi ,\eta )$. Then, the three images were used to calculate the binominal equations to retrieve the hyperspectrum raw data $SSS_0 (x_0,y_0;\lambda )$ from a captured photo $G(\xi ,\eta )$.

In the experiment, the measured spectral resolution is 10.1 nm. We compared the hyperspectral and CCT distribution measured by SISH with which measured by ODSG. Four types of LED packages were used as samples. It shows that for all types of packages, NCC values of the hyperspectral are higher than $95\%$ in the detection region $|\theta _T |<80^\circ$. AD and DSD calculated from the CCT curves within $|\theta _T |<60^\circ$ have the maximum values of 69k.

## Data Availability

The datasets used and/or analysed during the current study available from the corresponding author on reasonable request.
